# Syphilitic Condylomata of the Auditory Canal

**Published:** 1893-10

**Authors:** Charles Dunbar Roy

**Affiliations:** Professor Ophthalmology and Otology in Southern Medical College, Atlanta, Ga.


### SYPHILITIC CONDYLOMATA OF THE AUDITORY CANAL.*

By CHARLES DUNBAR ROY, A. B., M. D.,

Professor Ophthalmology and Otology in Southern Medical College, Atlanta, Ga.

Knowing as I did that this paper would be listened to almost entirely by those doing general medicine, it was my desire to present before the fellows of this society to-night a subject which would elicit thought and discussion from all, and in no wise be limited to those whose work lies in the narrow confines of specialism. That pathological condition known as syphilis is as extensive as the realm of medicine, and no subject, I am sure, even though it be limited to a special region of the human body, would call forth a more extended discussion from the members than this morbid condition save with the exception perhaps of some disease of the uterine appendages. Syphilis, as some writer has said, "is like the chain of memory, in that it links together the past and the present." It has left itself stamped even upon a race of prehistoric date if we may believe the appearances of bones exhumed in various parts of the earth, while even to-day it is no respector of persons. Writers tell us that the ancient literature of the Chinese and Japanese have shown works of great labor in the research of this subject, while its history to-day is seen in the general progress of medicine at large. But thanks to empiricism and scientific investigation of the latter day syphilographers, it is no longer considered the plague of the universe, nor the ravager of

*Read before Atlanta Society of Medicine, Sept. 19, 1893.

the body as it once was in days gone by. In fact, in Europe syphilis is no more thought of when infected than a sore throat or some other slight ailment. Yet it is not my purpose to go into a history of syphilis either from a social or a medical standpoint, but to bring before you to-night one of its manifestations as occurring in the auditory canal.

In the *Medical Record* of August 19th there appeared an article by Dr. Linthicum, of Baltimore on "Syphilis of the Auditory Canal with Report of a Case," and since the case in question was suggestive of one recently seen by myself, I shall take the liberty of presenting the same with some additional remarks.

C. M., colored, age twenty-six, presented himself at the Eye and Ear Clinic of the Southern Medical College in July of this year on account of some tenderness, discharge and difficulty of hearing in the left ear. He gave a history of syphilis, primary lesion, "breaking out," etc., dating back some two years previously. Over his left eyebrow could be seen an ulcerous condition, oval in shape, covered with a dirty scab. This had been present for a week. The acute trouble in the ear dated from four days previously, although there had been some discharge and difficulty in hearing for some months past. On examination the meatus was found to be almost entirely occluded by some swelling, and the presence of these warty excrescences which from the history and afterwards confirmed by the treatment, were diagnosed as syphilitic condylomata. Just behind the tragus there was marked tenderness which indicated an inflammatory process accompanied as it was by swelling. This was opened with some discharge of pus, and upon examination with a probe the sinus was found quite deep and dissecting. There was a slight dirty discharge, but not much more than one would expect from a subject morally opposed to ablutions. On account of the narrowed condition of the meatus the drum membrane could not be seen. The treatment consisted in painting the condylomata with a solution of nitrate of silver (20 gr. to ℥), the whole thoroughly touched with the yellow oxide of mercury ointment dusted afterwards with calomel. He was given the blue mercurial ointment to be rubbed on his brow together with the internal administration of the iodide of potash, and was ordered to keep the meatus cleaned with warm salt water. In two weeks' time under this *régime* the meatus had a smooth surface and its caliber was of normal dimensions, while the ulcerous condition of the brow had entirely disappeared. The membrana tympani was examined and gave no appearances of a previous involvement. Hearing was normal.

Syphilis is manifested in ear diseases, but by no means so frequently as in other portions of the body. For my statistics in regard to frequency of syphilis in ear diseases, I am indebted to the excellent article of Dr. J. Orne Green in Morrow's System of Genito-Urinary Diseases, etc., and to Prof. Politzer's Lehrbuch der Orenheilkunde, last edition. From the statistics published, it was found that "out of 1,370 syphilitics, thirteen, or 0.95 per cent., showed syphilis of the external ear; also that out of 7,515 patients suffering from ear disease, eighty-three, or 1.10 per cent., were subjects also of syphilis.

Primary syphilis of the ear is a very rare affection. During my term of service in Charity Hospital, New York, in a period covering eighteen months, I saw not a single case of this lesion, although hundreds of syphilitic patients were treated monthly, the amount of this material being enormous. Several cases of the primary lesion have been reported by various authors.

The cases of mediate infection in syphilis in contradistinction to the direct modes which have been reported by many syphilographers are much more infrequent to my mind than some writers would have us believe. In many of such cases it is my firm belief that the true history in the majority of cases has never been obtained by the physician himself. The lack of truthfulness in patients leads many a man to a false diagnosis.

Mracek in the Wiener Medical Presse, 1880, in a report of four hundred cases of chancres outside of the genital tract, gives but three of primary syphilis of the external ear. Prof. Gruber in the University at Vienna, having for a field of observation the combined clinics of Hebra and Von Sigmund, declares that he never saw a single case of the primary lesion on the ear. Cases have been reported by Zucker (Archives of Otology, 1884) and also by Hermet (Maladies de L'Oreille).

As far as I can gather only one case of this leison has been reported as occurring in the meatus itself. All the secondary lesions may occur on the auricle, but according to most observers the tubercular syphilides produce the most marked ulcerative changes. These secondary manifestations may occur in the cartilaginous portion of the external canal, and cases with the maculæ lesions occurring in this region have been reported by Urbantschitsch, and papules by Gruber, both observers being from Vienna.

Syphilitic ulcers, probably of late stages, have been reported by several observers. Condylomata of syphilitic origin located in the external auditory canal, and which have afforded the *raison d'être* for this article, have been found more frequently in this region than any other syphilitic manifestation.

Depres (Annales des Maladies de L'Oreille, 1878) out of twelve hundred syphilitic patients observed, nine hundred and eighty showed flat condylomata on different parts of the body, and of these five showed them in the meatus of the ear. Kipp in sixteen thousand ear patients saw condylomata of the meatus in two. In three thousand nine hundred and seventy-six ear patients Buck found five with condylomata in this region.

Condylomata in the meatus of the ear differ but little from those in other portions of the body, with the exception that they are mostly of the dry variety on account of their lack of contiguity to a mucous surface. Dr. Greene, of Boston, says that they have a greater tendency to ulceration, with the exception of those occurring in the genital regions, than on any other portion of the body. Their etiology is uncertain save of course its dependence upon a blood dyscrasia due to syphilis, the exciting cause evidently being due in a majority of cases to an acrid secretion. Mucous patches have been observed so constantly when condylomata occurred at the border of transition that one is almost obliged to believe that this occurrence is not entirely fortuitous.

Crocker, of London, says that a gonorrheal discharge is a frequent cause when they occur around the genitals.

Dr. Nevins Hyde, of Chicago, considers the condylomata as an altered form of the papuiar syphiloderm. He calls them vegetative mucous patches, usually presenting a moist, elevated surface which he thinks is no more than an overgrowth of the skin elements, consequent upon the irritative presence of secretion upon a papule. According to Knapp, of New York, there is first a diffused efflorescence in the canal, soon followed by swelling and the springing up of these elevated flat condylomatas of red, or gray-red, appearance. These warty excrescences are usually limited to just within the meatus, but Politzer has mentioned a case where the growths extended nearly to the drum membrane. According to Stöhr, pain sometimes occurs with the formation of these growths, and if they are of the ulcerative variety there are sometimes darting pains with the movement of the lower jaw. Sometimes there are subjunctive noises in the head and decrease in the hearing power, as occurred in the case I reported, these symptoms being due probably to a mechanical obstruction of the canal. On account of the irritating properties of these growths and their mechanical hinderance to the exit of the secretions normally occurring in the auditory canal, there is frequently a dirty grayish discharge filling every little crack and crevice and sometimes accompanied with a very foul odor. In the treatment of this affection cleanliness as the basis of all perfect treatment is of paramount importance. Every physician has his own ideas of treating syphilitic condylomata. I know of no better mode, or none more satisfactory in its result, than that pursued by me in treatment of the case reported. Here we possess an affection for the direct application of local and constitutional treatment.

56½ *Whitehall Street.*

---

THE latest reports show that cholera is very widely distributed throughout Europe, and is still slowly spreading. The disease exists in Russia, Bulgaria, Germany, Austria-Hungary, Galicia, the Netherlands, France, Belgium, Italy, Smyrna, and lastly, in Constantinople. As regards England, outbreaks of isolated cases have occurred at Grimsby and Hull, and, it is alleged, at Bradford, Sheffield, Rotherham and Belfast.—*Medical Record.*

